# Extracellular silica nanocoat formed by layer-by-layer (LBL) self-assembly confers aluminum resistance in root border cells of pea (*Pisum sativum*)

**DOI:** 10.1186/s12951-019-0486-y

**Published:** 2019-04-16

**Authors:** Yingming Feng, Xuewen Li, Shaoxue Guo, Xingyun Chen, Tingxuan Chen, Yongming He, Sergey Shabala, Min Yu

**Affiliations:** 1 Department of Horticulture, Foshan University, Foshan, 528000 Guangdong China; 2 Tasmanian Institute of Agriculture, University of Tasmania, Hobart, Australia

**Keywords:** Silica nanoparticle, Layer by layer self-assembly technique (LBL), Root border cells (RBCs), Mitochondrial activity, Aluminum toxicity

## Abstract

**Background:**

Soil acidity (and associated Al toxicity) is a major factor limiting crop production worldwide and threatening global food security. Electrostatic layer-by-layer (LBL) self-assembly provides a convenient and versatile method to form an extracellular silica nanocoat, which possess the ability to protect cell from the damage of physical stress or toxic substances. In this work, we have tested a hypothesis that extracellular silica nanocoat formed by LBL self-assembly will protect root border cells (RBCs) and enhance their resistance to Al toxicity.

**Results:**

Scanning electron microscopy (SEM) and X-ray photoelectron spectroscopy (XPS) were used to compare the properties of RBCs surface coated with nanoshells with those that were exposed to Al without coating. The accumulation of Al, reactive oxygen species (ROS) levels, and the activity of mitochondria were detected by a laser-scanning confocal microscopy. We found that a crystal-like layer of silica nanoparticles on the surface of RBCs functions as an extracellular Al-proof coat by immobilizing Al in the apoplast and preventing its accumulation in the cytosol. The silica nanoshells on the RBCs had a positive impact on maintaining the integrity of the plasma and mitochondrial membranes, preventing ROS burst and ensuring higher mitochondria activity and cell viability under Al toxicity.

**Conclusions:**

The study provides evidence that silica nanoshells confers RBCs Al resistance by restraining of Al in the silica-coat, suggesting that this method can be used an efficient tool to prevent multibillion-dollar losses caused by Al toxicity to agricultural crop production.

**Electronic supplementary material:**

The online version of this article (10.1186/s12951-019-0486-y) contains supplementary material, which is available to authorized users.

## Introduction

During the biological evolution, some marine organisms have developed various special structures to achieve optimal function. One of the obvious examples is a use of biominerals as their exterior coats/shells to fulfill many roles, including protection against physical stress and toxic substances [1–4]. Various (bio-) chemical modification methods have been developed to create (coat) artificial nanoshells on microbial or mammalian cells [5, 6]. It has also been shown that the nanoshells can confer cells new or unique properties [7–10]. Therefore, a material-based chemical strategy for the cell modification could meet various needs for more efficient applications of the cell biotechnology.

The electrostatic layer-by-layer (LBL) self-assembly provides a convenient and versatile method to synthesize nanoparticles with different surface compositions for strengthening the surface functionalization [2, 11–13]. The formation of a nanoparticle structure on the surface of solid is due to the alternating deposition of the polyelectrolyte by electrostatic interactions of anions and cations [3]. A uniformly thin and continuous layer of biocompatible silica is loaded on the surface of the yeast cell taking advantages of the LBL self-assembly technique [2]. Feifel et al. [14] demonstrates the potentiality of LBL to construct fully electro-active cyt *c* multilayer assemblies following natural examples of protein arrangements.

Silicon (Si) is taken up by roots mainly as monosilicic acid (H_4_SiO_4_) from the soil and translocated via xylem to the shoot. Although Si has not been considered as an essential element for higher plants, Si has been showed to play an astonishingly large number of diverse roles in various organisms [1]. Majority of Si is deposited in the specialized Si cells or extracellular space [15]. Intra- or extracellular solidification can significantly alleviate a range of abiotic stresses in rice plants, including heavy metal toxicity and lodging [16–18]. The presence of silicified structures improved plant cadmium tolerance of both rice seedlings and their single cells [16–18]. Silica nanostructures also enhance the capacity of plants or individual cell in coping with environmental stresses such as heat, drought stress and Cd toxicity [2, 10, 19].

Around 40% of the world’s arable land is acidic and thus contains high concentrations of free aluminum. Al hydrolyses in solution such that the trivalent Al species, Al^3+^, dominates in acid conditions (pH < 5) [20]. Thus, Al toxicity is a major limiting factor for plant growth and development in acid soils. While the breeding for Al tolerance has been on agenda for long time, most crops still display major yield losses when grown in acidic soils [21–23]. Can silica nanocoating ameliorate the impact of toxic Al species on root metabolism? To the best of our knowledge, no answers are available in the literature.

In this work, we have addressed this question by using LBL self-assembled nanoparticles for coating root border cells (RBCs), in an attempt to enhance their resistance to Al. Originated from the root cap meristematic cells, RBCs are programmed to produce and finally encapsulate the root apex. They are a population of the alive single cells that play a variety of biological functions in protecting the root tip from biotic and abiotic stress [24, 25]. Previous study shows RBCs function as a protection barrier of Al toxicity to root tips [25, 26], relying on the properties of cell wall in Al immobilization, predominantly in alkali-soluble pectin, which impairs Al access to the intracellular space [24, 27]. Our working hypothesis was that the LBL self-assembly of silica nanoparticles was supposed to enhance the roles of cell wall in Al immobilization as well as an “additional wall” of Al access to the symplast of RBCs. The findings of this work fully support this hypothesis and show that nanoshells formed on the surface of RBCs enhance its Al resistance by restraining the intrusion of Al into the cell, thus alleviating cellular injury.

## Materials and methods

### Plant materials

Seeds of pea (*Pisum sativum* L. cv Zhongwan no. 5) were germinated in a mist culture and RBCs were harvested as described previously [25, 28]. Pea seeds were immersed in 7.5% sodium hypochlorite for 30 min, and thereafter rinsed six times with deionized water. Seeds that were soaked, deformed and floating, were discarded, only unaltered seeds were kept and immersed in deionized water for 8 h, and then were spread on the mesh screen of the mist-culture device. Seeds were germinated at 24 °C in 20 L plastic tanks for 36 h with 80 s mist sprayed with 2 mM CaCl_2_ every 5 min. RBCs were harvested by snipping root tips into a plastic beaker containing 0.5 mM CaCl_2_ solution and stirring gently for 5 min. RBCs were pelleted at 4500×*g* for 10 min after removing the root tips. Pellets were re-suspended in an ultrapure water and centrifuged again. The rinsing procedure was repeated twice to yield purified RBCs.

### Silica mineralization on the surface of RBCs

The silica mineralization was modified following an established method [11]. RBCs were incubated in 2 μg/L PDADMAC (Poly dimethyl diallyl ammonium chloride, Sigma) solution (prepared in 1 mM/L CaCl_2_) for 10 min. Excessive PDADMAC was removed by centrifugation at 1000×*g* for 5 min, and the pelleted cells were rinsed three times in 1 mM/L CaCl_2_ solution. Pelleted cells were then reacted with PSS (Poly (sodium-*p*-styrenesulfonate), Sigma) for 10 min, and centrifuged and rinsed as described in PDADMAC loading. (PDADMAC/PSS)_2_ coated RBCs were obtained after repeating the process of PDADMAC/PSS loading for once. Then (PDADMAC/PSS)_2_ coated RBCs were incubated in 1% (w/v) silica particles (in 0.15 M NaCl) for 10 min, centrifuged and rinsed as described for PDADMAC loading.

### Cell viability of RBCs exposed to Al toxicity

Silicon coated or none-coated cells were exposed to 100 μM AlCl_3_ (2 mM CaCl_2_, pH 4.5) for 1 h. The cell viability was then detected microscopically by trypan blue (0.5%) exclusion test as described elsewhere [25].

### Al adsorption and Morin stain

After being treated with 100 μM AlCl_3_ solution (containing 2 mM CaCl_2_, pH 4.5) for 1 h, RBCs were pelleted at 4500×*g* for 10 min and surplus Al^3+^ in the supernatant was determined by pyrocatechol violet (PCV) following the methods described previously by Li et al. [27]. Cells were washed three times with deionized water, and then incubated in 0.01% Morin solution for 30 min and rinsed three times with de-ionized water. The green fluorescence signal was observed respectively using a laser-scanning confocal microscope (LSCM, FV1000, Olympus). The image analysis was performed using the ImageJ (https://rsb.info.nih.gov/ij).

### Scanning electron microscopy (SEM) and EDS analysis

After being treated with 100 μM AlCl_3_ solution (containing 2 mM CaCl_2_, pH 4.5) for 1 h, cells were pelleted at 4500×*g* and washed three times with 0.1 M PBS buffer solution. The pellet was then fixed in the glutaraldehyde (2.5%) solution for 24 h. Specimens were rinsed three times with 0.1 M PBS buffer and dehydrated through an ethanol dehydration series (30%, 50%, 70%, 90%, 95%) and 100% twice at 15-min intervals. The specimens were then processed with 1:1 (v/v) ethanol/isoamyl acetate mixed solution and isoamyl acetate solution for 10 min. Finally, specimens were desiccated in a critical-point dryer. SEM measurements (S-3700N, Hitachi, Japan) were performed for cell surface images, and the distribution of Ca and Al in cells was further analyzed by energy dispersive spectroscopy (EDS; Oxford, Inca 300, UK).

### X-ray photoelectron spectroscopy (XPS) studies

After being treated with 100 μM AlCl_3_ solution (containing 2 mM CaCl_2_, pH 4.5) for 1 h, RBCs were pelleted at 4500×*g* for 10 min and rinsed three times with deionized water, and finally the pelleted RBCs were freeze-dried and kept for analysis of surface element content by X-ray photoelectron spectroscopy [27]. RBCs samples were pressed onto plastic adhesive tape using a spatula to obtain a smooth surface for XPS measurement (VG multilab 2000 equipment Thermo VG scientific, East Grinstead, West Sussex, UK) using the Al Ka X-ray line of 1486.6 eV excitation energy at 300 W. RBCs were vacuum-dried for at least 8 h before the measurement. To correct sample charges, high-resolution spectra were used as a reference by setting the C1s hydrocarbon peak to 284.6 eV. The background was subtracted. Data analysis was performed using Thermal Advantage software (http://www.tainstruments.com) and draw by Originpro 9. The ratios of atomic concentrations were calculated using the peak areas normalized on a basis of acquisition parameters and sensitivity factors supplied by the manufacturer.

### Detection of reactive oxygen species (ROS)

Reactive oxygen species production was detected according to Hasanain et al. [29] with modifications. After Al^3+^ treatment for 1 h, the RBCs with or without nanoshells were incubated with 10 μM 5-(and 6)-chloromethyl-2′,7′-difluorodihydrofluorescein diacetate (CM-H_2_DCFDA, Thermo Fisher Scientific, Massachusetts, USA), dissolved in dimethyl sulfoxide and supplemented with 0.5 mM CaCl_2_, pH 4.5, in the dark at 25 °C for 30 min. Thereafter, the RBCs were rinsed with distilled water for 10 min in the dark. Fluorescence was imaged on a CLSM (FV1000, Olympus) at 488 nm excitation and 510–530 nm emission.

### Detection of mitochondrial activity and mitochondrial membrane potential

The mitochondrial activity of RBCs was assessed by MTT [3-(4, 5-dimethylthiazol-2)-2, 5-diphenyl-2*H*-tetrazolium bromide] reduction according to a previously published procedure [28]. Cells were pelleted at 4500×*g* for 10 min, rinsed three times with deionized water, and suspended in 0.5 mM CaCl_2_, containing 250 µg mL^−1^ MTT, (Thermo Fisher Scientific, Massachusetts, USA) before and after Al^3+^ treatment. The suspension was gently shaken at 25 °C for 2 h in the dark. The cells were then harvested, re-suspended in 5 mL of isopropanol containing 0.04 mM HCl, and vigorously mixed to dissolve the formazan produced from the cleavage of MTT. The cell pellets were removed via centrifugation, and the absorbance of formazan in the supernatant was determined spectrophotometrically at 590 nm. The MTT reduction of RBCs was expressed as formazan formation (A_590_/5 × 10^5^ cells).

The mitochondrial membrane potential was determined by JC-1 (5,5′,6,6′-tetrachloro-1,1′,3,3′-tetraethyl-benzimidazolyl-carbo-cyanine iodide, Thermo Fisher Scientific, Massachusetts, USA) staining. The purified cells were mixed with 20 μM JC-1 dye solution (1:1 [v/v]), and then incubated for 10 min at 37 °C. JC-1-loaded RBCs were excited at 488 nm using an Ar ion laser source, and the fluorescence was recorded simultaneously in two channels through a 515–545-nm band-pass filter (Green, Monomer) and a 580–600 nm long-pass filter (Red, J-aggregate) with two simultaneous independent detectors using a CLSM (FV1000, Olympus). Consequently, the mitochondrial depolarization is indicated by a decrease in the red/green fluorescence intensity ratio.

### Statistical analysis

All experiment was repeated independently for three times. Statistical analyses were performed using the statistical program SAS 9.4 and EXCEL 2013.

## Results

### The application of LBL self-assembly technique modifies surface properties of RBCs

**Fig. 1:**
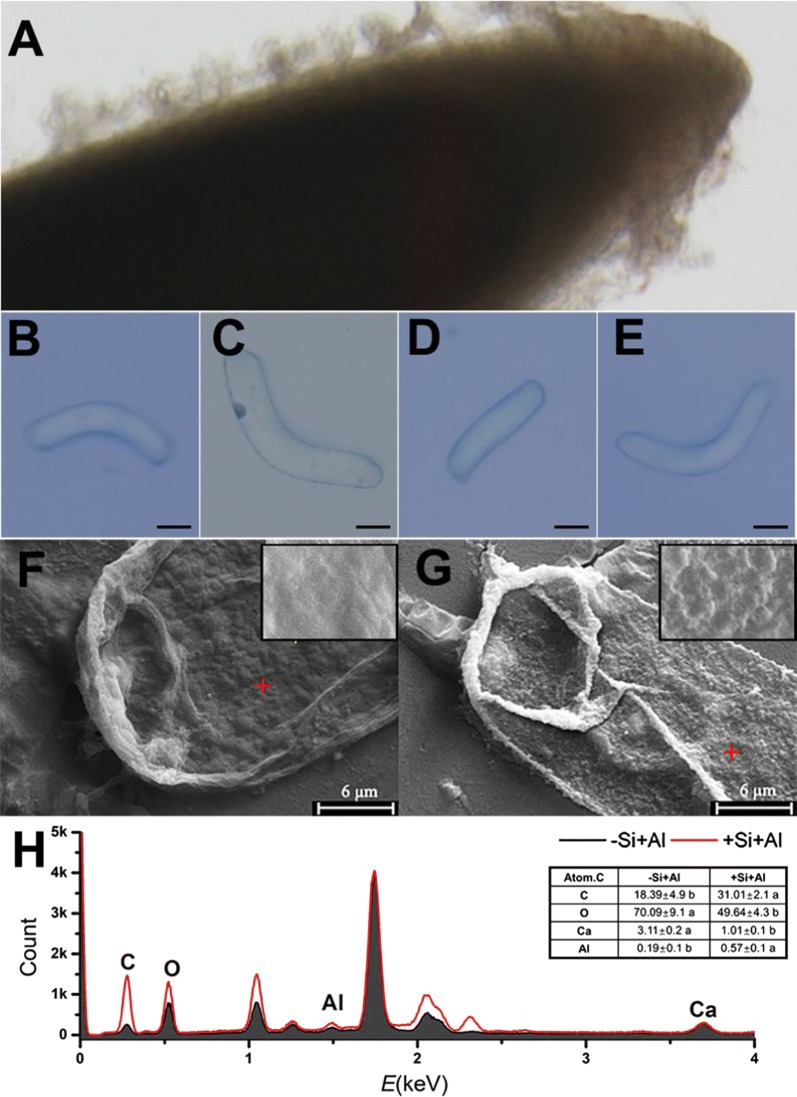
Properties of RBCs and scanning electron microscopy (SEM) image of the cells with or without the application of LBL self-assembly technique. RBCs are distributed around the root tip (**A**) and observed by trypan blue test. **B** Image of alive bare cell (−Si−Al); **C** Image of Al-induced dead bare cell exposed to 100 μM AlCl_3_ solution for 1 h (−Si+Al); **D** Image of silica-coat cell (+Si−Al); **E** Image of silica-coat cell exposed to 100 μM AlCl_3_ solution for 1 h (+Si+Al). Scale bars = 25 μm. RBCs was treated in 100 µM AlCl_3_ solution for 1 h, and then the specimens were dehydrated in ethanol and isoamyl acetate. SEM was performed for the surface images and the distribution of Ca and Al by energy dispersive spectroscopy. **F** Bare cells, **G** cells with silica-coat. Bar = 6 μm. Energy dispersive spectrometer (EDS) taken from the position of the red cross (**H**). The atomic content of carbon (C), oxygen (O), calcium (Ca) and aluminum (Al) was calculated

Root tips of pea and the attached RBCs grew vigorously in the mist culture (Fig. 1A); The trypan blue test was applied to observe the morphology and viability of silica-coated cells or bare cells exposed to 100 μM AlCl_3_ (pH 4.5) for 1 h (Fig. 1B–E). The harvested RBCs had width of 10 to 30 μm and were 100–200 μm in length. The exposure of Al induced cell death indicated in Fig. 1C. The images of SEM showed the differences in morphological features of RBCs with or without silica-coat under Al toxicity (Fig. 1F, G). The surface of the bare cells was relatively smooth, whereas the surface of cells treated by LBL technique became uneven, indicating the form of silica-coat. The analysis of EDS revealed that the cells with silica-coat accumulated more Al and less Ca compared with the bare cells (Fig. 1H). The ratios of C and O in cells treated by LBL self-assembly technique were also different from that of the bare cell. The results indicate the outermost layer of RBCs is covered with a unique new layer of silica nanoparticles which may enhance Al accumulation.

### Silica-coat promotes Al deposition in cell surface

**Fig. 2:**
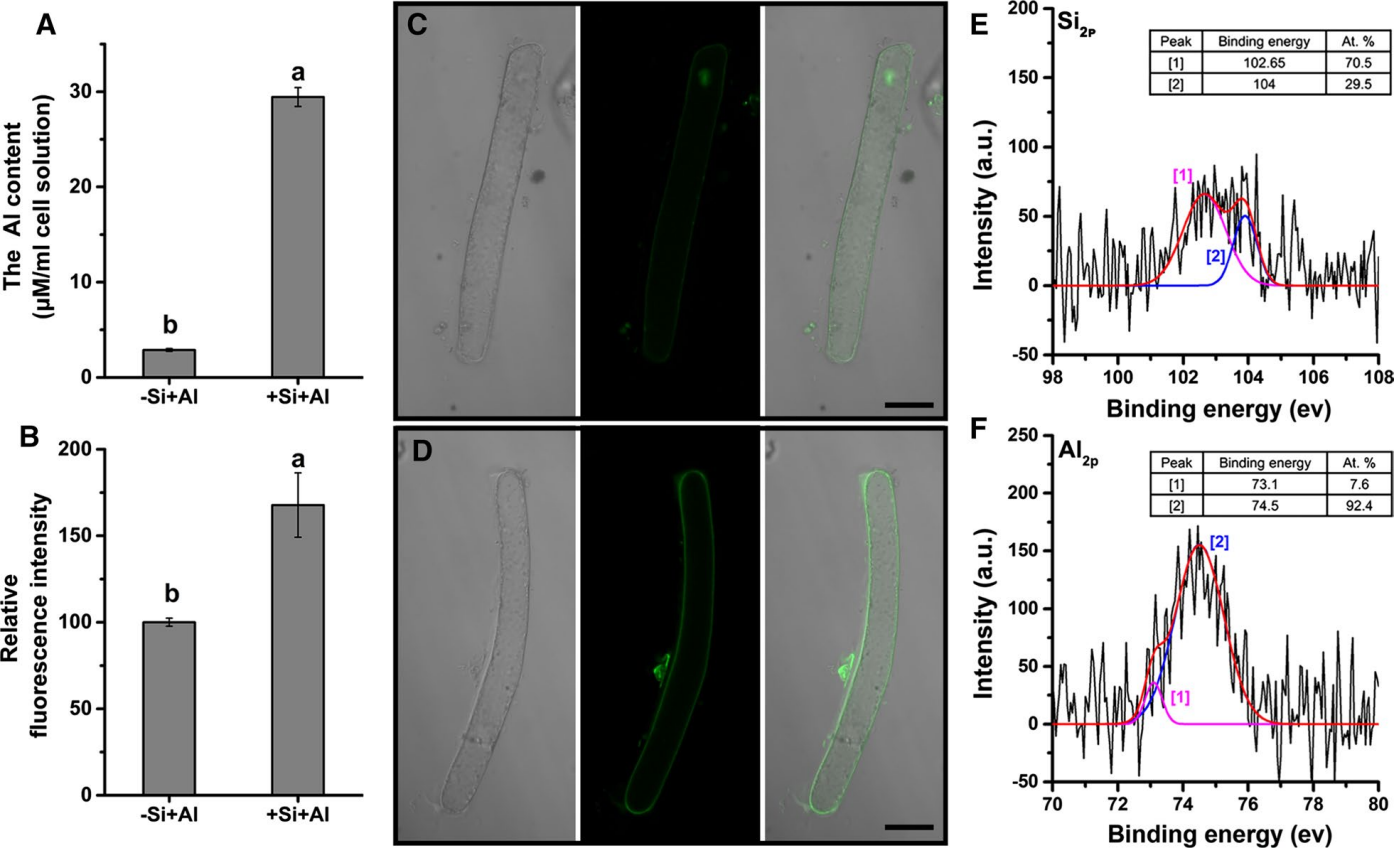
Al accumulation on the bare cells and silica-coated cells. RBCs were exposed to 100 µM AlCl_3_ solution at pH 4.5 for 1 h. **A** Al concentration in the solution was determined by the colorimetric method using pyrocatechol violet before and after the treatment, respectively, to deduce the amount of Al adsorption (n = 3). The distribution of Al in the bare cells (**C**) and the silica-coated cells (**D**) was observed using LSCM (n = 15). A relative fluorescence intensity of the Morin stain was calculated (**B**). Bar represents mean ± SE (n = 20). Different lowercase letters indicate significant differences at *p *< 0.05 between treatments (Duncan’s test). Scale bars = 25 μm. After Al treatment, RBCs with silica-coat (+Si–Al) were centrifuged and dried for X-ray photoelectron spectroscopy (XPS). The Si_2p_ (**E**) region was decomposed into two components at 102.65 and 104.0 eV, and the Al_2p_ (**F**) region was at 73.1 and 74.5 eV, suggesting the presence of aluminum silicate hydroxide at the cell wall surface [30, 31]

The adsorption assay showed that the silica-coated cells accumulated tenfold more Al than the bare cells (Fig. 2A), indicating an extraordinarily enhanced Al adsorption capacity of silica-coated cells. The stain of Morin also showed the surface of silica-coated cells accumulated more Al than the bare cells (Fig. 2B). Morin stain also disclosed the obvious Al accumulation in the nucleolus of bare cells (Fig. 2C, D), suggesting the intrusion of Al into the cells through the cell wall of RBCs without silica-coat.

The X-ray photoelectron spectroscopy (XPS) analysis was performed on the silica-coated cells for the information on the chemical composition of the cell surface. The Si_2p_ peak appeared near 102.65 and 104.0 eV (Fig. 2E) corresponding to aluminum silicate hydroxide [30] and typical of silica [31], respectively. The magnitude of the peak at ~ 102.65 eV (70.5 at %) was much higher than the components at 104.0 (29.5 at %). Al_2p_ peak appeared near ~ 73.10 eV (7.60 at %, Al_2_O_3_/Al [32]) and ~ 74.50 eV (92.4 at %, aluminum silicate hydroxide [30]) (Fig. 2F). These results indicated that Al on the surface of silica-coated cells mainly is mainly in the form of aluminum silicate hydroxide.

### Silica-coat inhibits ROS production in RBCs induced by Al

**Fig. 3:**
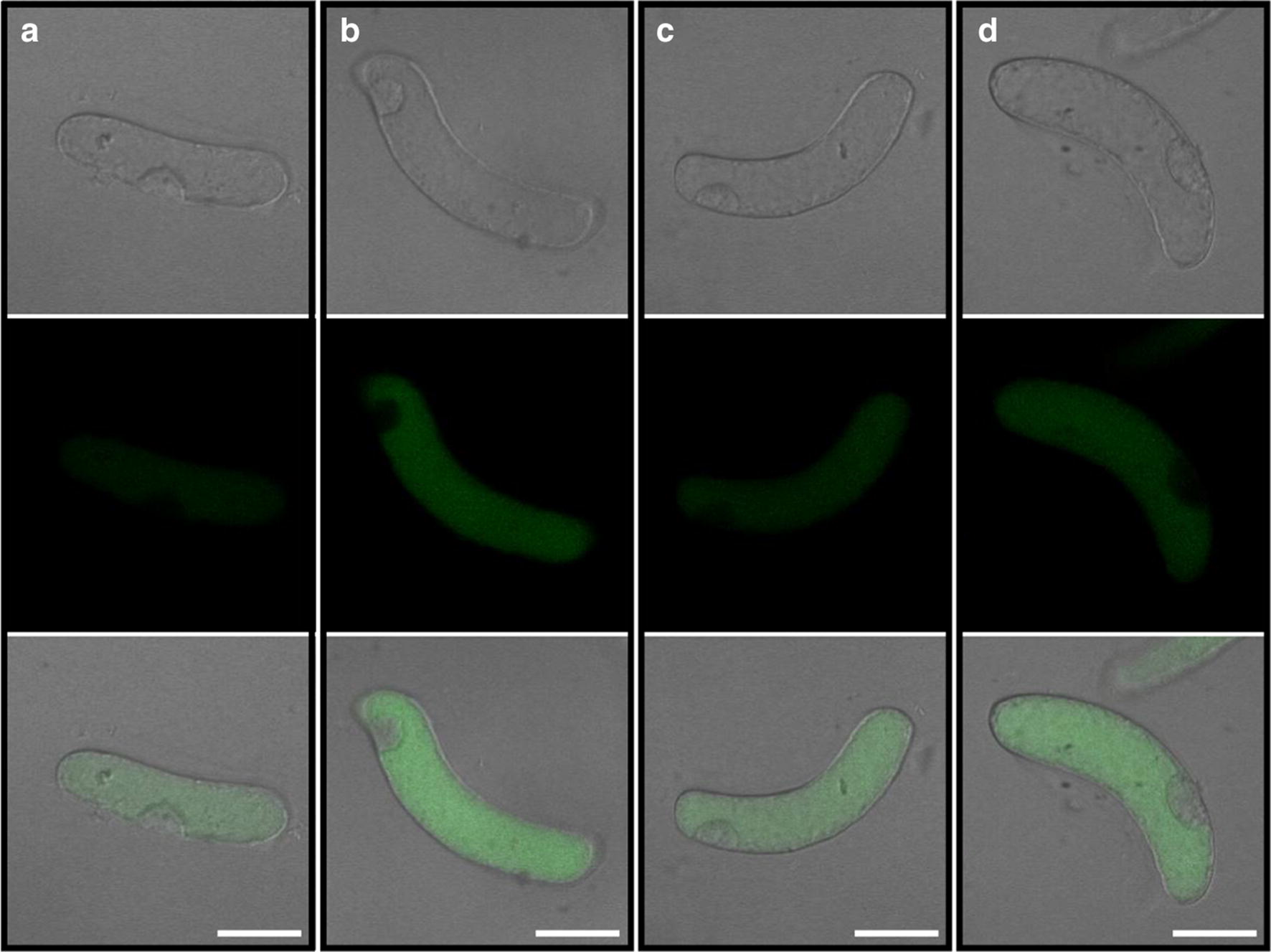
The production of ROS in RBCs. The cells were treated with 100 µM AlCl_3_ (pH 4.5) for 1 h. The probe of CM-H_2_DCFDA was used to estimate the production of ROS in bare cells and silica-coat cells using a CLSM (FV1000, Olympus). **a** Fluorescent and brightfield images of bare cells (−Si−Al); **b** Fluorescent and brightfield images of bare cells exposed to 100 μM AlCl_3_ solution for 1 h (−Si+Al); **c** Fluorescent and brightfield images of silica-coat cells (+Si–Al); **d** Fluorescent and brightfield images of silica-coat cells exposed to 100 μM AlCl_3_ solution for 1 h (+Si+Al). One (of 20) representative images is shown for each treatment. Scale bars = 25 μm

The fluorescent probe CM-H_2_DCFDA was applied to detect ROS in silica-coated cells or bare cells exposed to 100 μM AlCl_3_ (pH 4.5) for 1 h. In this method, the intensity of the green fluorescence signal is proportional to the amount of accumulated ROS. The results showed that Al toxicity induced significant ROS burst in bare cells (Fig. 3a, b). The coating of silica on the surface of RBCs induced a slightly increased ROS production in the silica-coated cells (Fig. 3c). However, Al induced less ROS production in silica-coated cells than in the bare cells under Al toxicity (Fig. 3c, d). Therefore, the results show that the nanoshells can reduce the intracellular ROS production caused by Al toxicity.

### Silica-coat enhances Al resistance of RBCs

**Fig. 4:**
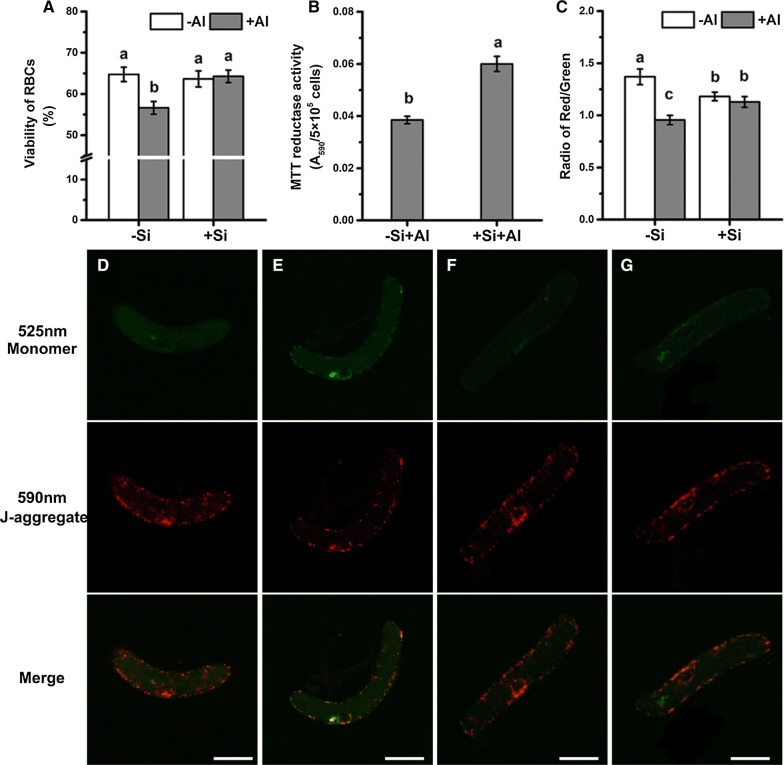
Cell viability and mitochondrial activity of RBCs. Cell viability of RBCs was measured by the trypan blue exclusion test (**A**). Mean ± SE (n = 10). A mitochondrial activity of RBCs was measured by the reduction of MTT after treatment with 100 μM AlCl_3_ (pH 4.5) for 1 h (**B**). Mean ± SE (n = 4). Mitochondrial membrane potential of RBCs was measured by JC-1 labelling. **D** Fluorescent images of JC-1 stained bare cells (−Si−Al); **E** Fluorescent images of JC-1 stained bare cells exposed to 100 μM AlCl_3_ solution for 1 h (−Si+Al); **F** Fluorescent images of JC-1 stained silica-coat cells (+Si−Al); **G** Fluorescent images of JC-1 stained silica-coat cells exposed to 100 μM AlCl_3_ solution for 1 h (+Si+Al). The intensity radio of red/green was calculated (**C**). Mean ± SE (n = 20). Scale bars = 25 μm. Different lowercase letters indicate significant differences at *p *< 0.05 (Duncan’s test)

Cell viability, determined by both trypan blue exclusion test and FDA-PI staining, decreased significantly after Al exposure in bare cells; however, this decrease was not observed in the silica-coated cells (Fig. 4A and Additional file 1: Figure S1). The results from the reduction of MTT also showed that the cells with nanoshells had a higher mitochondrial activity than the bare cells under Al toxicity (Fig. 4B), indicating less injuries occurred in mitochondria of cells with nanoshells.

The fluorescent probe JC-1 exhibit potential-dependent accumulation in mitochondria, indicated by a fluorescence emission shift from green (monomer, Em ~ 525 nm) to red (J-aggregate, Em ~ 590 nm), and mitochondrial depolarization is indicated by a decrease in the red/green fluorescence intensity ratio. The fluorescent probe JC-1 was used to specifically detect mitochondrial membrane potential of RBCs in here (Fig. 4D). The mitochondrial membrane potential decreased after bare cells were exposed to Al toxicity, as indicated by an increase in green fluorescence and radio of red/green (Fig. 4D, E). However, there was little differences in cells with nanoshells after Al exposure, although nano-coating process decreased the membrane potential (Fig. 4E, F). Therefore, membrane potential was significantly higher in the nanocoated cells than the bare cell under Al stress (Fig. 4C), which indicated that the nanocoated cell has stronger mitochondrial activity.

## Discussion

Layer-by-layer self-assembly technique can alternately deposit the anionic and cationic polyelectrolyte onto the solid surface by electrostatic interaction [33]. Meanwhile, LBL is a simple, abundant film-forming material, free from the base material of the body structure, and can protect the biologically active molecules, such as proteins and enzymes [11, 34, 35]. Through LBL self-assembly technique, the surface of RBCs became uneven (Fig. 1g), similar to the results on individual mammalian cells or yeast [2, 11]. That indicate LBL self-assembly technique can also form nanoshells on the surface of RBCs. Previous studies highlight the LBL self-assembly technique is a safe and non-toxic method [2, 36], which also been confirmed in this article. LBL self-assembly technique has little negative effect on the viability of RBCs (Fig. 4A, C), except inducing a slight increase in the ROS production (Fig. 3), which does not have to be a negative response [37–39].

Silica-coat confers the RBCs a peculiar capacity of Al adsorption from both EDS analysis of Al content in spots of single RBC and determination of Al absorption in millions of RBCs population. Cells covered by the silica nanoshells accumulated much more Al than the bare cells (Figs. 1G, 2A). Morin staining is generally used to study the Al distribution and semi-quantitative analysis of Al content [40]. Previous study proved that morin can detect Al in the cytosol but not cell wall-bound Al or vacuole-compartmentalized Al [41, 42]. After exposure to 100 μM Al for 1 h, surface of cells with silica nanoshells showed brighter green fluorescence compared with the bare cells (Fig. 2C, D), while nucleolus was brighter in the bare cells. Thus, it appears that the formation of nanoshells confers the RBCs an Al^3+^-proof “coat” that attracts Al in cell surface and blocks the entrance of Al^3+^ into the cell.

The images of SEM showed the surface of silica-coated cells became uneven and accompanied with small crystals (Fig. 1G). The thickness of the silica shell was 100 nm in yeast [2] or 200–300 nm in rice suspension cells [19]. XPS analysis can detect the surface elements down to a depth of 5–30 nm [43], which was used to analysis the surface chemical composition of the silica-coated RBCs. The Al_2p_ core-level X-ray photoelectron spectroscopy (XPS) spectra show an obvious peak at 74.5 eV (aluminum silicate hydroxide), Si_2p_ peak at 102.65 eV (aluminum silicate hydroxide), which indicated aluminum and silicon may form aluminum silicate hydroxides. The formation of Al–Si complexes, presumably hydroxyaluminosilicate species, leads to reduction of bioavailability of aluminum. Wang et al. [44] further showed the formation of hydroxyaluminumsilicates in the apoplast can reduces the toxicity of aluminum by reducing the mobility of apoplastic Al. Thus, the extra layer of silica provides sufficient protection as an Al-proof coat for RBCs, and inhibit the Al entering symplasts, preventing cell from ROS burst and a consequent injury.

The extra layer of the silica reduces the entry of Al^3+^ and accumulation of Al in symplast, preventing a burst of ROS [45, 46]. The accumulation of intracellular ROS will impact the integrity of the cellular membranes and organelles, e.g. mitochondria [47–49]. Adopting the trypan exclusion test as a proxy for membrane integrity, we found that nanocoated cells kept the integrity of membrane under Al exposure while the bare cells lost the membrane integrity (Fig. 4A). Morin stain detected little Al in the cytoplast of RBCs with nanoshells while obvious accumulation of Al in nucleolus of bare cells was observed. Since less Al enters the RBCs with silica-coat (Fig. 2), relatively less ROS production was induced by Al toxicity as indicated by ROS probe (Fig. 3). The accumulation of ROS leads to the imbalance of intracellular redox homeostasis [50, 51]. Our work showed that silica nano-coat on the surface of RBCs reduces the production of ROS induced by Al toxicity, which allows cells to maintain higher activity under Al toxicity.

Al-triggered ROS production is considered to be a cause of ATP depletion [48, 52]. The accumulation of ROS in cells and the imbalance of intracellular redox homeostasis will lead to depolarization of the mitochondrial membrane potential and diminished mitochondrial activity [48, 53]. This may be a result of direct damage caused by ROS to the membrane system [54, 55], or be a result of ROS activation of ion transporters in mitochondrial membranes [56, 57]. The resultant mitochondrial dysfunction will then lead to the programed cell death (PCD) [58–60]. The membrane potential was significantly higher in nanocoated cells than the bare cells under Al stress (Fig. 4D, F). The mitochondrial membrane potential decreased after bare cells were exposed to Al toxicity (Fig. 4B). RBCs coated with silica had a higher mitochondrial activity than the bare cells after exposing to Al toxicity (Fig. 4A and Additional file 1: Figure S1). Therefore, silica-coated cells maintained higher membranes potential, enabling normal activity of mitochondria under Al toxicity, and preventing their PCD.

**Fig. 5:**
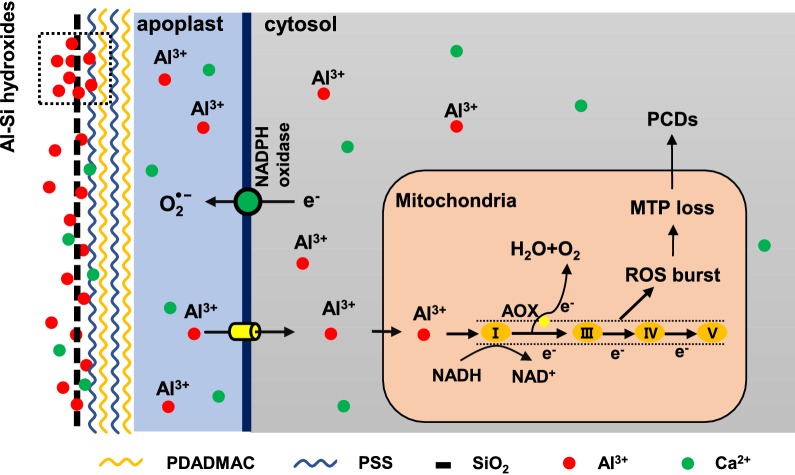
The proposed working model for the silica coating enhances the Al resistance of the RBCs. LBL deposition of PDADMAC/PSS onto the RBCs surface induced the formation of silica nanoparticles layer, which prevents Al^3+^ into the symplast by forming aluminum silicate hydroxides (the dotted box). Reactive oxygen species (ROS) can be generated from the mitochondrial electron transport system through electron leaks when substrates are metabolized. Reducing Al^3+^ accumulation in mitochondria prevents ROS burst and a consequent loss of the mitochondrial transmembrane potential (MTP). As a result, Al-induced mitochondria-dependent program cell death (PCD) is alleviated

Root border cells act as a phalanx of biological ‘goalies’, which neutralize dangers to newly generated root from the soil. As summarized in the model (Fig. 5), silica nanoparticles layer, formed by LBL self-assembly, confers RBCs Al resistance by binding Al and forming aluminum silicate hydroxides, thus inhibits the entry of Al^3+^ into the symplast, therefore preventing ROS burst and injury to the mitochondria. These findings have major translational implications, by offering an approach for improvement of plant tolerance to Al toxicity caused by acid soils that occupies 40% of the land, thus ensuring the sustainability of agriculture and contributing to the global food security in the twenty first century.

## Additional file

**Additional file 1: Figure S1.** Cell viability was measured by FDA-PI staining. RBCs were exposed to 100 µM AlCl_3_ solution at pH 4.5 for 1 h, and cell viability was determined by FDA-PI staining. In brief, RBCs were stained for 10 min with a mixture of FAD (12.5 μg/mL)-PI (5 μg/mL) solution, then cells were observed with a fluorescence microscope (Olympus IX71) under blue light excitation (510 nm). Mean ± SE (n = 5). Different lowercase letters indicate significant differences at *p *< 0.05 (Duncan’s test).
