# Supplementary figures and images for: Extracellular silica nanocoat formed by layer-by-layer (LBL) self-assembly confers aluminum resistance in root border cells of pea (Pisum sativum)
Source: J Nanobiotechnology. 2019 Apr 16;17:53. doi: 10.1186/s12951-019-0486-y (PMC6466759; doi:10.1186/s12951-019-0486-y)

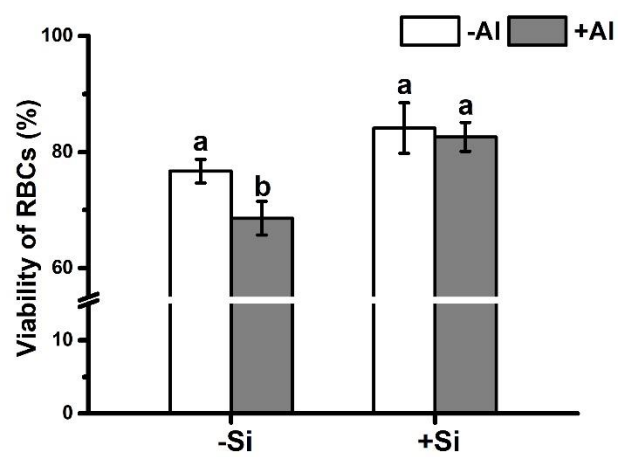

Supplement: Supplementary file 1 — Additional file 1: Figure S1. Cell viability was measured by FDA-PI staining. RBCs were exposed to 100 µM AlCl3 solution at pH 4.5 for 1 h, and cell viability was determined by FDA-PI staining. In brief, RBCs were stained for 10 min with a mixture of FAD (12.5 μg/mL)-PI (5 μg/mL) solution, then cells were observed with a fluorescence microscope (Olympus IX71) under blue light excitation (510 nm). Mean ± SE (n = 5). Different lowercase letters indicate significant differences at p < 0.05 (Duncan’s test). [file 12951_2019_486_MOESM1_ESM.pdf]
